# (*E*)-4-Chloro­benzyl 3-(3-nitro­benzyl­idene)dithio­carbazate

**DOI:** 10.1107/S1600536809047953

**Published:** 2009-11-14

**Authors:** Huan-Qiu Li, Yin Luo, Dong-Dong Li, Hai-Liang Zhu

**Affiliations:** a State Key Laboratory of Pharmaceutical Biotechnology, Nanjing University, Nanjing 210093, People’s Republic of China

## Abstract

In the title compound, C_15_H_12_ClN_3_O_2_S_2_, the dihedral angle between the aromatic rings is 89.71 (10)°. In the crystal, inversion dimers linked by pairs of N—H⋯S hydrogen bonds occur.

## Related literature

For background to the chemistry of carbodithio­ates, see: Tarafder *et al.* (2002 ▶).
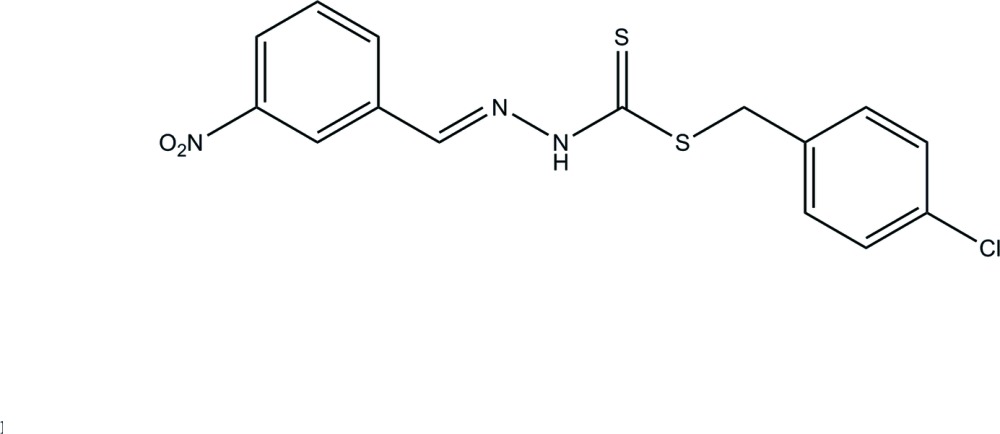


## Experimental

### 

#### Crystal data

C_15_H_12_ClN_3_O_2_S_2_
*M* *_r_* = 365.85
Monoclinic, 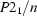

*a* = 10.175 (2) Å
*b* = 8.4958 (17) Å
*c* = 19.318 (4) Å
β = 105.01 (3)°
*V* = 1613.0 (6) Å^3^
*Z* = 4
Mo *K*α radiation
μ = 0.51 mm^−1^
*T* = 293 K
0.25 × 0.15 × 0.15 mm

#### Data collection

Enraf–Nonius CAD-4 diffractometer
Absorption correction: ψ scan (North *et al.*, 1968 ▶) *T*_min_ = 0.884, *T*_max_ = 0.928
10135 measured reflections
3086 independent reflections
2538 reflections with *I* > 2σ(*I*)
*R*_int_ = 0.019
200 standard reflections every 3 reflections intensity decay: 1%

#### Refinement

*R*[*F* ^2^ > 2σ(*F* ^2^)] = 0.032
*wR*(*F* ^2^) = 0.086
*S* = 1.08
3086 reflections
212 parameters
H atoms treated by a mixture of independent and constrained refinement
Δρ_max_ = 0.49 e Å^−3^
Δρ_min_ = −0.44 e Å^−3^

### 

Data collection: *CAD-4 Software* (Enraf–Nonius, 1989 ▶); cell refinement: *CAD-4 Software*; data reduction: *XCAD4* (Harms & Wocadlo, 1995 ▶); program(s) used to solve structure: *SHELXS97* (Sheldrick, 2008 ▶); program(s) used to refine structure: *SHELXL97* (Sheldrick, 2008 ▶); molecular graphics: *SHELXTL* (Sheldrick, 2008 ▶); software used to prepare material for publication: *SHELXTL*.

## Supplementary Material

Crystal structure: contains datablocks global, I. DOI: 10.1107/S1600536809047953/hb5222sup1.cif

Structure factors: contains datablocks I. DOI: 10.1107/S1600536809047953/hb5222Isup2.hkl

Additional supplementary materials:  crystallographic information; 3D view; checkCIF report

## Figures and Tables

**Table 1:** Hydrogen-bond geometry (Å, °)

*D*—H⋯*A*	*D*—H	H⋯*A*	*D*⋯*A*	*D*—H⋯*A*
N1—H1⋯S2^i^	0.83 (2)	2.73 (2)	3.4565 (18)	147.7 (18)

Symmetry code: (i) 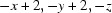.

**Fig. 1.**
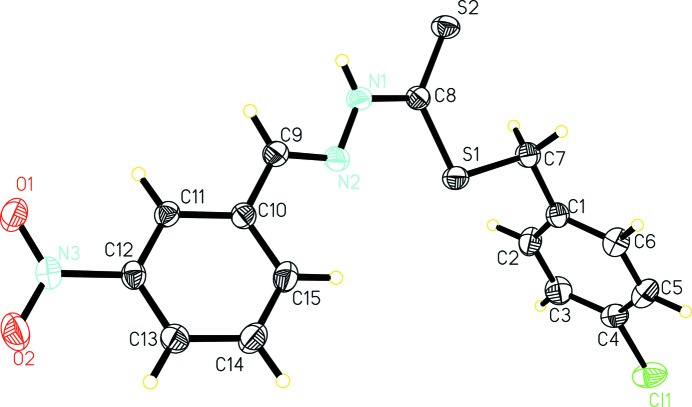
The structure of (I) showing 50% displacement ellipsoids.

## supplementary crystallographic information

### Experimental

A mixture of benzyl hydrazinecarbodithioate (396 mg, 2 mmol), 3-nitrobenzaldehyde (302 mg, 2 mmol) was stirred in methanol (10 ml) for 1 h. After keeping the filtrate in air for 7 d, yellow blocks of (I) were formed.

### Refinement

The H atoms were positioned geometrically (C—H = 0.93–0.96 Å) and refined as riding, with *U*_iso_(H) = 1.2*U*_eq_(C).

### Crystal data

**Table:**

C_15_H_12_ClN_3_O_2_S_2_	*F*(000) = 752
*M**_r_* = 365.85	*D*_x_ = 1.507 Mg m^−^^3^
Monoclinic, *P*2_1_/*n*	Mo *K*α radiation, λ = 0.71073 Å
Hall symbol: -P 2yn	Cell parameters from 25 reflections
*a* = 10.175 (2) Å	θ = 9–12°
*b* = 8.4958 (17) Å	µ = 0.51 mm^−^^1^
*c* = 19.318 (4) Å	*T* = 293 K
β = 105.01 (3)°	Block, yellow
*V* = 1613.0 (6) Å^3^	0.25 × 0.15 × 0.15 mm
*Z* = 4	

### Data collection

**Table:**

Enraf–Nonius CAD-4 diffractometer	2538 reflections with *I* > 2σ(*I*)
Radiation source: fine-focus sealed tube	*R*_int_ = 0.019
graphite	θ_max_ = 26.0°, θ_min_ = 2.1°
ω/2θ scans	*h* = −12→11
Absorption correction: ψ scan (North *et al.*, 1968)	*k* = −10→10
*T*_min_ = 0.884, *T*_max_ = 0.928	*l* = −23→23
10135 measured reflections	200 standard reflections every 3 reflections
3086 independent reflections	intensity decay: 1%

### Refinement

**Table:**

Refinement on *F*^2^	Primary atom site location: structure-invariant direct methods
Least-squares matrix: full	Secondary atom site location: difference Fourier map
*R*[*F*^2^ > 2σ(*F*^2^)] = 0.032	Hydrogen site location: inferred from neighbouring sites
*wR*(*F*^2^) = 0.086	H atoms treated by a mixture of independent and constrained refinement
*S* = 1.08	*w* = 1/[σ^2^(*F*_o_^2^) + (0.0407*P*)^2^ + 0.5179*P*] where *P* = (*F*_o_^2^ + 2*F*_c_^2^)/3
3086 reflections	(Δ/σ)_max_ = 0.003
212 parameters	Δρ_max_ = 0.49 e Å^−^^3^
0 restraints	Δρ_min_ = −0.44 e Å^−^^3^

### Special details

**Table:**

Geometry. All e.s.d.'s (except the e.s.d. in the dihedral angle between two l.s. planes) are estimated using the full covariance matrix. The cell e.s.d.'s are taken into account individually in the estimation of e.s.d.'s in distances, angles and torsion angles; correlations between e.s.d.'s in cell parameters are only used when they are defined by crystal symmetry. An approximate (isotropic) treatment of cell e.s.d.'s is used for estimating e.s.d.'s involving l.s. planes.
Refinement. Refinement of *F*^2^ against ALL reflections. The weighted *R*-factor *wR* and goodness of fit *S* are based on *F*^2^, conventional *R*-factors *R* are based on *F*, with *F* set to zero for negative *F*^2^. The threshold expression of *F*^2^ > σ(*F*^2^) is used only for calculating *R*-factors(gt) *etc*. and is not relevant to the choice of reflections for refinement. *R*-factors based on *F*^2^ are statistically about twice as large as those based on *F*, and *R*- factors based on ALL data will be even larger.

### Fractional atomic coordinates and isotropic or equivalent isotropic displacement parameters (Å^2^)

**Table:**

	*x*	*y*	*z*	*U*_iso_*/*U*_eq_	
C1	0.88396 (19)	0.8024 (2)	0.29108 (9)	0.0425 (4)	
C2	0.9588 (2)	0.6812 (3)	0.33047 (11)	0.0549 (5)	
H2	1.0335	0.6410	0.3170	0.066*	
C3	0.9240 (2)	0.6188 (3)	0.38966 (11)	0.0590 (5)	
H3	0.9749	0.5375	0.4158	0.071*	
C4	0.8134 (2)	0.6784 (3)	0.40920 (10)	0.0508 (5)	
C5	0.7369 (2)	0.7970 (3)	0.37079 (11)	0.0565 (5)	
H5	0.6619	0.8364	0.3843	0.068*	
C6	0.7724 (2)	0.8578 (3)	0.31159 (10)	0.0509 (5)	
H6	0.7198	0.9376	0.2851	0.061*	
C7	0.9279 (2)	0.8774 (2)	0.23026 (9)	0.0481 (5)	
H7A	1.0261	0.8896	0.2433	0.058*	
H7B	0.8873	0.9810	0.2206	0.058*	
C8	0.96762 (18)	0.8426 (2)	0.09548 (9)	0.0379 (4)	
C9	0.8344 (2)	0.6100 (2)	−0.05521 (9)	0.0435 (4)	
H9	0.8960	0.6424	−0.0804	0.052*	
C10	0.72835 (18)	0.49645 (19)	−0.08815 (9)	0.0377 (4)	
C11	0.74117 (18)	0.41334 (19)	−0.14820 (8)	0.0364 (4)	
H11	0.8164	0.4281	−0.1664	0.044*	
C12	0.63997 (18)	0.30863 (19)	−0.18004 (8)	0.0363 (4)	
C13	0.52623 (19)	0.2843 (2)	−0.15556 (10)	0.0442 (4)	
H13	0.4591	0.2138	−0.1784	0.053*	
C14	0.5144 (2)	0.3672 (3)	−0.09628 (10)	0.0515 (5)	
H14	0.4385	0.3524	−0.0786	0.062*	
C15	0.6144 (2)	0.4724 (2)	−0.06269 (10)	0.0480 (5)	
H15	0.6051	0.5275	−0.0226	0.058*	
Cl1	0.77017 (6)	0.60320 (8)	0.48441 (3)	0.0716 (2)	
H1	0.974 (2)	0.822 (3)	−0.0005 (11)	0.050 (6)*	
N1	0.94398 (17)	0.77726 (19)	0.03009 (8)	0.0448 (4)	
N2	0.84407 (16)	0.66553 (17)	0.00735 (8)	0.0423 (4)	
N3	0.65325 (16)	0.22357 (18)	−0.24405 (8)	0.0425 (4)	
O1	0.74879 (15)	0.25400 (16)	−0.26884 (7)	0.0530 (4)	
O2	0.56589 (15)	0.12601 (19)	−0.27048 (8)	0.0667 (4)	
S1	0.87573 (5)	0.75559 (6)	0.15042 (2)	0.04588 (15)	
S2	1.07801 (5)	0.98879 (6)	0.11887 (2)	0.04813 (15)	

### Atomic displacement parameters (Å^2^)

**Table:**

	*U*^11^	*U*^22^	*U*^33^	*U*^12^	*U*^13^	*U*^23^
C1	0.0481 (11)	0.0450 (10)	0.0339 (8)	−0.0057 (8)	0.0095 (8)	−0.0079 (8)
C2	0.0586 (13)	0.0574 (12)	0.0536 (11)	0.0094 (10)	0.0232 (10)	−0.0032 (10)
C3	0.0710 (15)	0.0550 (12)	0.0506 (12)	0.0065 (11)	0.0152 (10)	0.0067 (9)
C4	0.0549 (12)	0.0612 (12)	0.0362 (9)	−0.0174 (10)	0.0114 (8)	−0.0053 (9)
C5	0.0443 (11)	0.0792 (15)	0.0484 (11)	−0.0008 (11)	0.0166 (9)	−0.0039 (11)
C6	0.0464 (11)	0.0605 (12)	0.0442 (10)	0.0040 (9)	0.0091 (8)	0.0001 (9)
C7	0.0577 (12)	0.0491 (11)	0.0389 (9)	−0.0120 (9)	0.0149 (8)	−0.0094 (8)
C8	0.0423 (10)	0.0372 (9)	0.0337 (8)	−0.0012 (7)	0.0089 (7)	0.0011 (7)
C9	0.0534 (11)	0.0424 (10)	0.0369 (9)	−0.0059 (8)	0.0156 (8)	−0.0009 (8)
C10	0.0447 (10)	0.0361 (9)	0.0327 (8)	0.0001 (7)	0.0105 (7)	0.0009 (7)
C11	0.0401 (10)	0.0376 (9)	0.0330 (8)	0.0016 (7)	0.0119 (7)	0.0030 (7)
C12	0.0417 (10)	0.0353 (9)	0.0312 (8)	0.0050 (7)	0.0082 (7)	0.0000 (7)
C13	0.0412 (10)	0.0468 (10)	0.0437 (10)	−0.0042 (8)	0.0094 (8)	−0.0050 (8)
C14	0.0455 (11)	0.0659 (13)	0.0485 (11)	−0.0049 (10)	0.0218 (9)	−0.0070 (9)
C15	0.0534 (12)	0.0546 (11)	0.0402 (10)	−0.0017 (9)	0.0197 (8)	−0.0091 (9)
Cl1	0.0795 (4)	0.0922 (5)	0.0438 (3)	−0.0314 (3)	0.0171 (3)	0.0014 (3)
N1	0.0569 (10)	0.0442 (9)	0.0360 (8)	−0.0148 (7)	0.0169 (7)	−0.0038 (7)
N2	0.0525 (9)	0.0395 (8)	0.0354 (7)	−0.0078 (7)	0.0124 (7)	−0.0027 (6)
N3	0.0437 (9)	0.0431 (8)	0.0388 (8)	0.0061 (7)	0.0073 (7)	−0.0050 (7)
O1	0.0554 (9)	0.0634 (9)	0.0457 (7)	0.0008 (7)	0.0231 (7)	−0.0067 (6)
O2	0.0573 (9)	0.0734 (10)	0.0703 (10)	−0.0139 (8)	0.0183 (7)	−0.0364 (8)
S1	0.0549 (3)	0.0488 (3)	0.0358 (2)	−0.0159 (2)	0.0151 (2)	−0.00558 (19)
S2	0.0555 (3)	0.0466 (3)	0.0414 (3)	−0.0161 (2)	0.0111 (2)	−0.0018 (2)

### Geometric parameters (Å, °)

**Table:**

C1—C6	1.379 (3)	C9—N2	1.277 (2)
C1—C2	1.385 (3)	C9—C10	1.464 (2)
C1—C7	1.503 (3)	C9—H9	0.9300
C2—C3	1.387 (3)	C10—C15	1.387 (3)
C2—H2	0.9300	C10—C11	1.393 (2)
C3—C4	1.373 (3)	C11—C12	1.379 (2)
C3—H3	0.9300	C11—H11	0.9300
C4—C5	1.369 (3)	C12—C13	1.375 (3)
C4—Cl1	1.745 (2)	C12—N3	1.468 (2)
C5—C6	1.385 (3)	C13—C14	1.376 (3)
C5—H5	0.9300	C13—H13	0.9300
C6—H6	0.9300	C14—C15	1.383 (3)
C7—S1	1.8186 (18)	C14—H14	0.9300
C7—H7A	0.9700	C15—H15	0.9300
C7—H7B	0.9700	N1—N2	1.377 (2)
C8—N1	1.343 (2)	N1—H1	0.83 (2)
C8—S2	1.6571 (18)	N3—O1	1.218 (2)
C8—S1	1.7495 (18)	N3—O2	1.226 (2)
			
C6—C1—C2	118.19 (18)	N2—C9—H9	119.3
C6—C1—C7	120.93 (18)	C10—C9—H9	119.3
C2—C1—C7	120.79 (18)	C15—C10—C11	119.20 (16)
C1—C2—C3	121.03 (19)	C15—C10—C9	122.19 (16)
C1—C2—H2	119.5	C11—C10—C9	118.59 (16)
C3—C2—H2	119.5	C12—C11—C10	118.58 (16)
C4—C3—C2	119.2 (2)	C12—C11—H11	120.7
C4—C3—H3	120.4	C10—C11—H11	120.7
C2—C3—H3	120.4	C13—C12—C11	122.78 (16)
C5—C4—C3	120.97 (19)	C13—C12—N3	119.02 (16)
C5—C4—Cl1	119.35 (17)	C11—C12—N3	118.17 (15)
C3—C4—Cl1	119.68 (17)	C12—C13—C14	118.20 (17)
C4—C5—C6	119.2 (2)	C12—C13—H13	120.9
C4—C5—H5	120.4	C14—C13—H13	120.9
C6—C5—H5	120.4	C13—C14—C15	120.57 (18)
C1—C6—C5	121.4 (2)	C13—C14—H14	119.7
C1—C6—H6	119.3	C15—C14—H14	119.7
C5—C6—H6	119.3	C14—C15—C10	120.67 (17)
C1—C7—S1	109.93 (13)	C14—C15—H15	119.7
C1—C7—H7A	109.7	C10—C15—H15	119.7
S1—C7—H7A	109.7	C8—N1—N2	121.50 (16)
C1—C7—H7B	109.7	C8—N1—H1	118.1 (15)
S1—C7—H7B	109.7	N2—N1—H1	118.0 (15)
H7A—C7—H7B	108.2	C9—N2—N1	115.23 (15)
N1—C8—S2	120.64 (14)	O1—N3—O2	123.12 (15)
N1—C8—S1	113.72 (13)	O1—N3—C12	118.93 (15)
S2—C8—S1	125.62 (10)	O2—N3—C12	117.94 (16)
N2—C9—C10	121.48 (17)	C8—S1—C7	100.92 (8)
			
C6—C1—C2—C3	−1.0 (3)	C11—C12—C13—C14	−0.7 (3)
C7—C1—C2—C3	175.56 (18)	N3—C12—C13—C14	−178.84 (17)
C1—C2—C3—C4	0.1 (3)	C12—C13—C14—C15	0.2 (3)
C2—C3—C4—C5	0.6 (3)	C13—C14—C15—C10	0.1 (3)
C2—C3—C4—Cl1	−178.96 (16)	C11—C10—C15—C14	0.0 (3)
C3—C4—C5—C6	−0.3 (3)	C9—C10—C15—C14	178.26 (18)
Cl1—C4—C5—C6	179.27 (16)	S2—C8—N1—N2	−174.10 (14)
C2—C1—C6—C5	1.4 (3)	S1—C8—N1—N2	7.3 (2)
C7—C1—C6—C5	−175.24 (18)	C10—C9—N2—N1	−176.76 (16)
C4—C5—C6—C1	−0.7 (3)	C8—N1—N2—C9	−178.30 (17)
C6—C1—C7—S1	−102.99 (19)	C13—C12—N3—O1	174.50 (16)
C2—C1—C7—S1	80.5 (2)	C11—C12—N3—O1	−3.7 (2)
N2—C9—C10—C15	16.4 (3)	C13—C12—N3—O2	−4.7 (2)
N2—C9—C10—C11	−165.39 (17)	C11—C12—N3—O2	177.13 (16)
C15—C10—C11—C12	−0.5 (2)	N1—C8—S1—C7	−177.81 (14)
C9—C10—C11—C12	−178.76 (15)	S2—C8—S1—C7	3.71 (15)
C10—C11—C12—C13	0.8 (3)	C1—C7—S1—C8	−167.97 (14)
C10—C11—C12—N3	178.97 (14)		

### Hydrogen-bond geometry (Å, °)

**Table:**

*D*—H···*A*	*D*—H	H···*A*	*D*···*A*	*D*—H···*A*
N1—H1···S2^i^	0.83 (2)	2.73 (2)	3.4565 (18)	147.7 (18)

Symmetry codes: (i) −*x*+2, −*y*+2, −*z*.
